# Wide-field auroral imager onboard the Fengyun satellite

**DOI:** 10.1038/s41377-019-0157-7

**Published:** 2019-05-22

**Authors:** Xiao-Xin Zhang, Bo Chen, Fei He, Ke-Fei Song, Ling-Ping He, Shi-Jie Liu, Quan-Feng Guo, Jia-Wei Li, Xiao-Dong Wang, Hong-Ji Zhang, Hai-Feng Wang, Zhen-Wei Han, Liang Sun, Pei-Jie Zhang, Shuang Dai, Guang-Xing Ding, Li-Heng Chen, Zhong-Su Wang, Guang-Wei Shi, Xin Zhang, Chao Yu, Zhong-Dong Yang, Peng Zhang, Jin-Song Wang

**Affiliations:** 1 National Satellite Meteorological Center, China Meteorological Administration, Beijing, China; 2 Key Laboratory of Space Weather, National Center for Space Weather, China Meteorological Administration, Beijing, China; 3 Changchun Institute of Optics, Fine Mechanics and Physics, Chinese Academy of Sciences, Changchun, China; 4 State Key Laboratory of Applied Optics, Chinese Academy of Sciences, Changchun, China; 5 Key Laboratory of Earth and Planetary Physics, Institute of Geology and Geophysics, Chinese Academy of Sciences, Beijing, China; 6 Institutions of Earth Science, Chinese Academy of Sciences, Beijing, China

**Keywords:** Atmospheric optics, Imaging and sensing

## Abstract

The newly launched Fengyun-3D (FY-3D) satellite carried a wide-field auroral imager (WAI) that was developed by Changchun Institute of Optics, Fine Mechanics and Physics, Chinese Academy of Sciences (CIOMP), which will provide a large field of view (FOV), high spatial resolution, and broadband ultraviolet images of the aurora and the ionosphere by imaging the N_2_ LBH bands of emissions. The WAI consists of two identical cameras, each with an FOV of 68° in the along-track direction and 10° in the cross-track direction. The two cameras are tilted relative to each other to cover a fan-shaped field of size 130° × 10°. Each camera consists of an unobstructed four-mirror anastigmatic optical system, a BaF_2_ filter, and a photon-counting imaging detector. The spatial resolution of WAI is ~10 km at the nadir point at a reference height of 110 km above the Earth’s surface. The sensitivity is >0.01 counts s^−1^ Rayleigh^−1^ pixel^−1^ (140–180 nm) for both cameras, which is sufficient for mapping the boundaries and the fine structures of the auroral oval during storms/substorms. Based on the tests and calibrations that were conducted prior to launch, the data processing algorithm includes photon signal decoding, geometric distortion correction, photometric correction, flat-field correction, line-of-sight projection and correction, and normalization between the two cameras. Preliminarily processed images are compared with DMSP SSUSI images. The agreement between the images that were captured by two instruments demonstrates that the WAI and the data processing algorithm operate normally and can provide high-quality scientific data for future studies on auroral dynamics.

## Satellite imaging: Mapping Earth’s auroras for space weather

A compact camera system on board China’s recently launched Fengyun-3D satellite can reveal subtle features of the Earth’s auroras for improved space weather forecasting. Auroras such as the northern lights arise when solar wind charged particles precipitate along the Earth’s magnetic field and collide with atmospheric gases. Xiao-Xin Zhang, Bo Chen and their colleagues have developed a high-resolution imager that can help researchers predict geomagnetic substorms and storms from the dynamic configurations of auroras. The imager uses two cameras to simultaneously scan auroral regions from a low Earth orbit, delivering an expanded field of view relative to previous instruments. In-orbit testing shows that the device’s mirror-based optics and data processing system can identify boundaries and structures of auroras with 10 kilometer-scale resolution.

## Introduction

The wide-field auroral imager (WAI) is designed for studying the dynamics of auroras and potentially for forecasting auroral substorms by imaging the N_2_ Lyman–Birge–Hopfield (LBH) auroral bands. The LBH aurora is generated by the collision with the molecular nitrogen of the energetic electron precipitating into the polar upper atmosphere along the Earth’s magnetic field lines^1,2^. The intensity of the auroral LBH emissions depends on the flux and mean energy of the precipitated electrons and the mixed auroral boundary. Typically, the auroral oval is located at magnetic latitudes between 65° and 75°, however, it expands to lower latitudes at nightside during storms and substorms. Imaging the auroral oval from space provides spatial/temporal information of the precipitation particles that can be mapped back to different source regions in the magnetosphere to elucidate the energy transportation processes^3,4^. Space-borne optical remote sensing of the aurora has been under development for more than 40 years. Many imagers and spectrographs that are sensitive in various spectral bands (e.g., X-ray, extreme ultraviolet (EUV), far ultraviolet (FUV), and visible light) have been launched to study auroral phenomena. Among these, two categories of instruments have been commonly used.

The first category consists of multispectral imagers that operate in elliptical polar orbits, such as the scanning auroral imager (SAI) that is onboard the Dynamic Explorer 1 (DE-1) satellite^5^, the wideband imaging camera (WIC) and spectrographic imager (SI) that are onboard the Imager for Magnetosphere-to-Auroral Global Exploration (IMAGE) spacecraft^6–8^, and the ultraviolet imager that is onboard the Polar satellite^9^. The instruments typically operate in the FUV wavelength range and global auroral images with various emission features (typically at H 121.6 nm, OI 130.4 nm, OI 135.6 nm, N_2_ LBH bands) have typically been obtained when the satellites were located in apogee regions that are more than 30,000 km from the Earth. In such scenarios, the complete auroral oval can be observed at a spatial resolution of ~100 km and the images can only be used to investigate the large-scale structures of the aurora.

The second category of instruments, which operate in low-Earth orbits, can realize higher spatial resolution (~50 km) but with small spatial coverage and the complete auroral oval cannot be observed in a snapshot. These instruments include the global ultraviolet imager (GUVI) that is onboard the Thermosphere Ionosphere Mesosphere Energy and Dynamics (TIMED) mission^10^ and the special sensor ultraviolet spectrographic imager (SSUSI) that is onboard the Defense Meteorological Satellite Program (DMSP) satellites^11^. The two instruments are almost identical, except the altitude is ~630 km for GUVI and ~840 km for SSUSI. Both imagers measure the spectral radiance of the Earth’s FUV airglow and aurora in the spectral range from 120 to 180 nm using a cross-track scanning spectrometer design. Once the spectral information of the airglow and auroral emissions are obtained, various environmental parameters can be derived, such as the column O/N_2_ ratio^12^, the mixed auroral boundaries, the effective energy flux, the effective average energy, and the ionospheric conductivities^3,4^. A summary of the parameters of these typical imagers is presented in Table 1.

**Table 1:** Summary of typical auroral imagers

Imager	Angular/Spatial Resolution^a^ (deg/km)	Spacecraft altitude	Wavelength (nm)	Image frame rate	Reference
WAI/FY-3D	0.8/10	830 km	140–180	2, 100 min	This paper
WIC/IMAGE	0.18/120	0.3–7 *R*_E_	140–190	10 s	^6, 7^
SI/IMAGE	0.13/100 0.26/200	0.3–7 *R*_E_	135.6 121.6	5 s	^8^
UVI/Polar	0.03/30	1–8 *R*_E_	4 filters: 130–190	37 s	^9^
GUVI/TIMED	0.8/50	630 km	Spectrometer: 120–180	100 min	^10^
SSUSI/DMSP	0.8/50	840 km	Spectrometer: 120–180	100 min	^11^
SAI/DE-1	0.32/100	1–4 *R*_E_^b^	Several filters: 121.6–630.0	12 min	^5^

^a^The spatial resolution refers to the resolution of the projected disk images

^b^1 *R*_E_ = 6375.0 km, which is the Earth’s radius

Benefiting from the observations by the above imagers (and possibly other imagers), many large-scale auroral structures were discovered in the past decades^13–17^, which led to many discoveries of physical and chemical processes that occur in the magnetosphere but are not fully visible to any in-situ instruments. It is frequently stated that “the small auroral oval is a projection of the vast magnetosphere–ionosphere system”. Therefore, the small structures of the auroral oval could be the results of large-scale processes in the magnetosphere. With these concerns, we have developed a state-of-the-art spacecraft-based imager that features both high resolution and a wide field for monitoring the auroral dynamics from a low-Earth orbit.

In this paper, we will identify the requirements of WAI according to the scientific goals and the constraints that are associated with the FY-3 satellite platform. The design and development of the WAI instrument, the laboratory tests and calibrations of WAI, the data processing algorithms, and the preliminary results will be presented in detail.

## Results

**Fig. 1:**
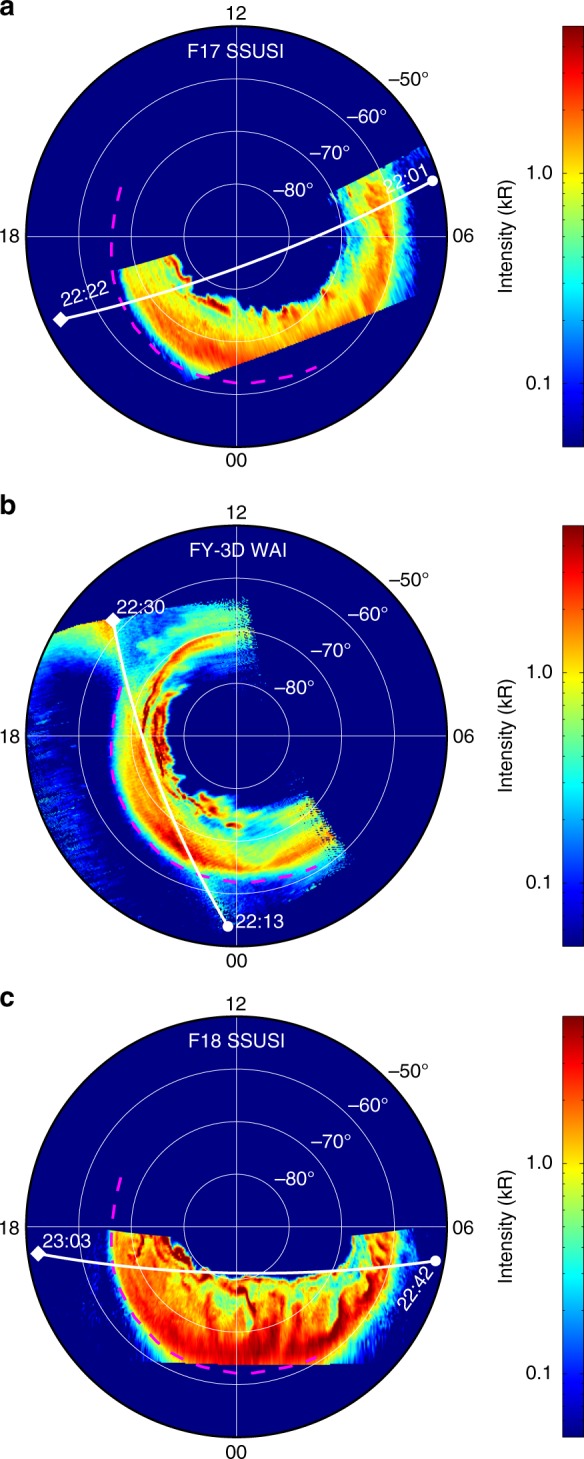
Auroral images that were captured by FY-3D WAI and DMSP SSUSI in the southern hemisphere on August 25, 2018. **a** The crossing of DMSP F17 in the southern hemisphere from 22:01 UT to 22:22 UT. **b** The crossing of FY-3D in the southern hemisphere from 22:13 UT to 22:30 UT. **c** The crossing of DMSP F18 in the southern hemisphere from 22:42 UT to 23:03 UT. The pink dashed lines represent the auroral boundary that was extracted from the FY-3D WAI image

Figure 1 shows the auroral images that were captured from 22:01 UT to 23:03 UT on August 25, 2018 by WAI (in nadir mode) and SSUSI during the expansion phase of a geomagnetic storm. The DMSP F17 satellite passed the southern polar region from 22:01 UT to 22:22 UT, the FY-3D satellite passed the southern polar region from 22:13 UT to 22:30 UT, and the DMSP F18 satellite passed the polar region from 22:42 UT to 23:03 UT. A threshold of 50 Rayleigh is applied to all three images to eliminate the background noises. According to Fig. 1, the auroral image that was captured by WAI corresponds very well with those that were captured by F17 and F18; hence, the WAI instrument and the data processing algorithm perform well. From Fig. 1a–c, equatorward expansion and intensification of the auroral oval are observed. Therefore, auroral imaging with both high temporal resolution and high spatial resolution is necessary. In addition, according to Fig. 1b, the spatial coverage of WAI is significantly larger than that of DMSP SSUSI; hence, we can investigate the auroral dynamics in a larger spatial scope and evaluate the magnetosphere-ionosphere coupling in a wider region. Moreover, since the F17, FY-3D, and F18 satellites always pass the polar region successively with a temporal interval of ~20 min, combination of the three satellites could provide more comprehensive temporal and spatial coverage in auroral researches in the future.

The result in Fig. 1b is preliminary. The extended structure in the upper-left corner in Fig. 1b is caused by the dayglow of the ionosphere. In the current version of the data processing algorithm, such noises are preserved. We will investigate the application of a new background removal algorithm in the future to eliminate these noises.

## Discussion

In this paper, the development, calibration, and data processing algorithms for the WAI onboard FY-3 satellite are described. This is the first optical remote sensor for space weather and space physics in China. The results of in-orbit tests demonstrate that the performance of WAI satisfies the design requirements and the requirements of scientific investigations. The processed Level-1 disk images of the aurora and ionosphere will be released by the NCSW-CMA according to its data policy. Another high-level product is under development.

Auroral morphology is a powerful diagnostic tool for investigating energy dissipation processes in the Earth’s magnetospheres. The basic driver of auroral explosions is the interaction of the solar wind with the Earth’s magnetosphere; however, the understanding of the physical processes that lead to various types of auroras is incomplete. Auroral explosions can be global or local, depending on the global dynamics of the magnetospheric plasma transportation and the flux and energy of the plasma. Therefore, an auroral imager with both a large field and high resolution is beneficial for investigating auroras both globally and locally. When FY-3H is launched in the near future, two WAIs will be operating in a same orbit and the auroral activities in both hemispheres could be monitored simultaneously. We will be able to investigate the auroral dynamics and solar wind–magnetosphere–ionosphere couplings, including their hemispheric differences, systematically. This will also substantially improve our understanding of space weather and its accurate prediction.

## Materials and methods

### Scientific objectives

As the official space weather operation agency, the National Center for Space Weather, China Meteorological Administration (NCSW-CMA) is responsible for planning China’s space weather observation infrastructures. The concept of wide-field auroral imaging was initially proposed by Chinese scientists in the KuaFu mission^18^, which was designed to explore the physical processes that are responsible for space weather as a complement to planned in situ and ground-based programs and to make an essential contribution to the space weather applications. In 2012, the NCSW-CMA proposed carrying two WAIs on the polar-orbiting Fengyun-3 satellites, namely, the FY-3D and FY-3H satellites, to simultaneously monitor the ionosphere and the auroral activity in both hemispheres.

Both FY-3D and FY-3H will operate in a polar-orbiting sun-synchronous orbit at an altitude of ~840 km with ~99° inclination and with orbital periods near 100 min. An orbital phase difference of 180° will be set for the two satellites to ensure the simultaneous observations of auroral oval in both hemispheres with high spatial resolution and large spatial coverages. Such conjugated auroral imaging is highly important for investigating the auroral breakup, the auroral polar boundary intensification, which is due to far magnetotail reconnection, and the cusp auroral, which is generated by the high-latitude lobe reconnection under conditions of northward interplanetary magnetic field (IMF). Using conjugated imaging, we can also conduct research on the hemispheric symmetry and asymmetry properties in the energy input to the geospace system (especially the ionosphere). By determining the conductivity difference at the foot points of the magnetic flux tubes in both hemispheres, the effect of the conductivity in driving the substorm expansion phase and other dynamic processes can be derived. Global conjugated imaging can also facilitate the determination of when the diffuse auroral and discrete auroral images become conjugated.

WAI will also observe the LBH emissions in the dayside ionosphere in middle and low latitudes to capture the large-scale structures and evolutions of the ionosphere and thermosphere. The airglow emissions are excited mostly by collisions of photoelectrons that are generated by solar extreme ultraviolet radiation with the major gases in the upper atmosphere. The brightness of the LBH emissions can be used as a measure of the integrated solar flux^12^, which is a proxy for the energy flux into the atmosphere in a variable region of the solar spectrum.

The main scientific objectives for WAI are as follows:

1. Image the LBH aurora in the spectral range from 140 to 180 nm to monitor and potentially forecast the auroral substorms.
2. Identify the small-scale structures of the aurora in the FUV wavelength on the time scale of minutes to investigate the physical and chemical processes in the source regions in the transition regions between the inner and outer magnetosphere.
3. Image the LBH emission in the middle- and low-latitude ionosphere to capture the large-scale structures and evolutions of the ionosphere and thermosphere.

Based on these objectives and considering the orbital properties of the FY-3 satellite, the WAI system should have the following characteristics:

1. A spectral response that is limited to the N_2_ LBH band.
2. Optical system(s) with large FOV and high resolution.
3. High sensitivity to ensure a short exposure time.

According to these requirements, the WAI instrument parameters are summarized in Table 2. The design will be analyzed in detail in the following sections.

**Table 2:** FY-3WAI requirement summary

Parameter	Value
Wavelength	140–180 nm (FY-3D)
	140–160 nm, 160–180 nm (FY-3F)
Field of view (instantaneous)	130° (cross-track) × 10° (along-track)
Along-track sweeping range	±60° from the nadir
Angular resolution	0.8° (10 km at the nadir in disk images)
Sensitivity	>0.01 counts s^−1^ Rayleigh^−1^ pixel^−1^ (140–180 nm)
Nadir pointing accuracy	<0.16°
Image frame rate	<2 min for scanning mode
	~100 min for nadir mode

### Optical and mechanical design

**Fig. 2:**
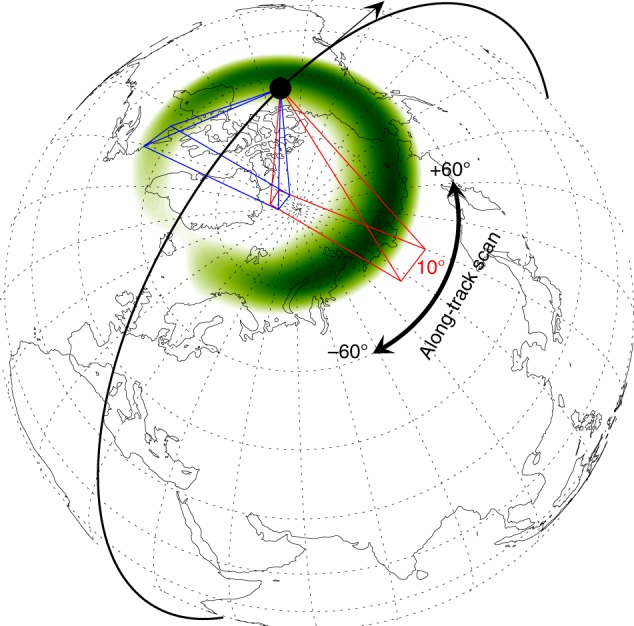
Illustration of the operation of the WAI instrument. The auroral oval is represented in green. The thick black arc represents the orbit of FY-3D. The red and blue lines represent the FOVs of the two cameras

The spatial resolution and FOV of WAI depend not only on the satellite orbit but also on the optical system design. The FY-3 satellites operate in a near-circular orbit at a height of ~840 km. Suppose that the auroral images are projected on a reference sphere at a height of 110 km. The angle between the nadir and the tangential line to the reference sphere is ~65° for an arbitrary point on the orbit, as illustrated in Fig. 2. Therefore, the maximum FOV needed for disk imaging is 130° in both the along-track and cross-track directions. However, it is almost impossible to realize an instantaneous FOV of 130° × 130° for the reflective optical system since the transmissive optical system cannot be used in the FUV range. A more suitable method for realizing such a large FOV is to assemble several small identical FOVs.

Considering the convenience of optical system alignment and the limitations on the weight and size of WAI that are imposed by the satellite platform, the design is as follows: The WAI consists of two identical cameras that are served by common electronics. Each camera has a 68° × 10° FOV and the two cameras are tilted relative to each other by 65° to cover a fan-shaped instantaneous FOV of 130° × 10°, namely, 130° in the cross-track direction and 10° in the along-track direction, as illustrated in Fig. 2. For such a large FOV, the optimal angular resolution of 0.8° can be realized. At a height of 840 km, the corresponding spatial resolution on a reference sphere of 110 km in height is 10 km at the nadir. As the satellite moves, the fan sweeps along-track to form a 130° × 130° coverage area, namely, the sweeping range is 120° along-track. This design can realize the largest spatial coverage at a height of ~840 km, except for special cases when sun avoidance occurs in the sunlit polar region.

**Fig. 3:**
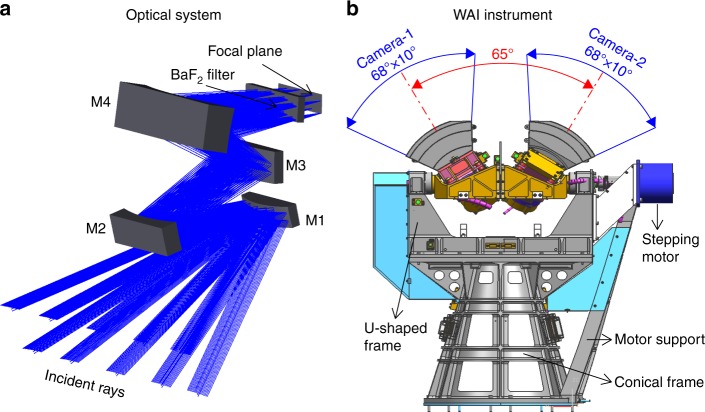
Overview of the WAI instruments. **a** The off-axial astigmatic optical system of WAI. **b** The mechanical design of the WAI

An off-axial astigmatic optical system is designed for each camera, as illustrated in Fig. 3a, which consists of four mirrors (M1, M2, M3, and M4), a temperature control filter, and a photon-counting imaging detector. The optical system has the following characteristics: (1) the “inverted telephoto”^19^ four-mirror optical system, in which the first mirror is a convex mirror, realizes a large FOV of 68° × 10°; (2) the noncoaxial layout of the optical system improves the imaging quality and yields superior stray light suppression; (3) all the mirrors are spherical and, thus, are easy to fabricate and align; and (4) the focal plane is flat, with no Pezval field curvature, namely, the sum of the curvatures of the four mirrors equals zero. The optical system of each camera of the WAI has an FOV of 68° × 10°, an angular resolution of better than 0.8°, a focus of 18.5 mm and an entrance aperture area of 0.22 cm^2^.

Since the main scientific objective of WAI is to monitor the auroral activities, two main operation modes are designed: The first is the scanning mode, which is utilized at high latitudes if the magnetic latitude of the satellite exceeds 60° in both hemispheres. The scanning mode includes forward scanning (along the fly direction) and backward scanning (opposite the fly direction). The sweeping range in both directions is 120° with 60° at the nadir. To investigate the auroral dynamics on the time-scale of minutes, the sweeping period is designed to be less than 2 min. The motor will drive the headers to sweep a range of 120° in ~107 s with an angular speed of 1.125°/s. Every sweep is divided into several frames, each of which is composed of several exposures. According to the angular resolution, the rotating speed of the head, and the line speed of the satellite nadir, the accumulation time for each exposure is set to 0.34 s to ensure that the equivalent pixel shift on the detector is less than half a pixel. The second mode is the nadir mode. The FOV points to the satellite nadir and remains still. The accumulation time for each exposure is also 0.34 s.

By default, each frame contains 6 exposures, which is equivalent to an accumulation time of 2.04 s for a fixed pixel of size 10 km by 10 km on the reference sphere. In addition, the pointing accuracy and the pointing stability must be better than 0.16° (less than 1/5 pixel) and 0.16°/s, respectively, to ensure accurate pixel projection and image merging in the scientific data processing algorithm. The pointing accuracy of the FY-3D satellite is better than 0.15° and the pointing stability is better than 0.009°/s, which is sufficient for the imaging of WAI.

According to the requirements of science and optical design, the structure of WAI should be compact and lightweight. The two cameras are accurately mounted together and can sweep a range of 120° in 2 min. The whole structure should be stable and long-lived in the space environment. The designed mechanical structure of the WAI is illustrated in Fig. 3b, which is composed of two cameras that are symmetrically mounted on a single frame, a U-shaped frame that supports the frame, a precision stepper motor for rotating the cameras, a base support and an additional support. To minimize the weight of the rotating parts, reduce the height of the center of mass and improve the structural stability, the rotating parts only include the frame and two cameras and the center of mass of the rotating parts has been adjusted to coincide with the axis. The electronic cables from the two cameras are laid through the inner hole of the shaft to minimize the damage that is caused by the rotation of the mechanism. The base support is used to elevate the cameras. It is ensured that the stray light that is produced by the satellite body and other instruments cannot contaminate the optical system. The stepper motor rotates the cameras over a range of 120° at a speed of 1.125°/s to realize auroral imaging of a 130° × 130° area in two min. There are three prisms on the frame and the cameras for calibrating the directions of the axes.

### Spectra response

**Fig. 4:**
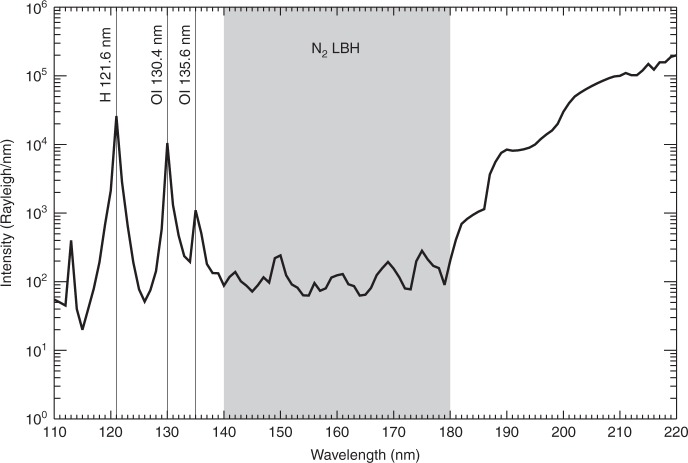
Auroral spectrum of 1-nm resolution with the WAI passband overlaid. The shaded region corresponds to the response wavelength of WAI

Figure 4 shows a typical auroral spectrum in the FUV range^1,20^. There are several auroral emission lines in the FUV range, e.g., H Ly-α at 121.6 nm, OI 130.4 nm, OI 135.6 nm, and the N_2_ LBH bands. The dayglow spectrum lines in the middle- and low-latitude ranges are similar to those in Fig. 4; however, they differ in terms of the intensity distribution. Many instruments, especially spectrographic imagers (e.g., GUVI and SSUSI), can simultaneously observe all these lines in the auroral region and the ionosphere. Only the photons in the response range of the instrument can be recorded by the detectors. Due to the spatial and weight limits on the satellite, especially on FY-3D, WAI must be designed to be highly compact. There is insufficient space for including a complex wavelength selection assembly (such as that in Polar UVI) in WAI. Therefore, WAI was designed to be a single-channel imager and only the LBH band (140–180 nm) was selected for WAI on FY-3D. On the following FY-3H, the LBH band will be divided into two channels, namely, 140–160 nm (LBHS) and 160–180 nm (LBHL), to enable the electron flux and the mean energy of the precipitating electrons to be obtained by inverting the relationship between the ratios of the emissions.

To ensure accurate inversion of the observed emission intensity, the most important indicator of an imager is the spectral response. The instrument must have a sufficiently high response in its passband and a sufficiently low out-of-band response. As shown in Fig. 4, the auroral emissions at Ly-α, 130.4 nm and 135.6 nm are 1–2 orders of magnitude above the LBH emission and the intensity increases at least 2 orders of magnitude above 180 nm. Most auroral imaging of the polar atmosphere will be conducted when the solar illumination is at a slant angle and the dayglow contribution is substantially reduced. For the typical spectrum in Fig. 4, the out-of-band response of WAI should be 5% of the response in 140–180 nm. To satisfy this requirement, three designs should be considered.

The first is a narrowband pass response of the mirrors of the optical system. Multilayer coating technology can realize this. The shorter and longer cutoff wavelengths of the mirror coatings should be set at 140 and 180 nm, respectively. The cutoff edge cannot be too sharp for maintaining a sufficiently high reflectivity in the passband. A quarter-wave 150 nm multilayer was superposed to a quarter-wave 170 nm multilayer and this initial multilayer structure was further optimized via the constrained optimization function of the OptiLayer software. The average reflectance of the mirror is 48.6% and the reflectance is lower than 15% at wavelengths that are shorter than 137 nm or longer than 187 nm.

The second is a high-pass filter for further suppressing the responses that are shorter than 140 nm. This filter should have very low transmission at wavelengths that are shorter than 140 nm and uniform and high transmission at wavelengths that exceed 140 nm. The barium fluoride (BaF_2_) crystal is the optimal choice for this filter. At room temperature, the transmission of BaF_2_ is less than 1% at wavelengths that are shorter than ~134 nm and higher than 70% at wavelengths that are longer than 140 nm. An important feature of the transmission property of BaF_2_ is the drift of the cutoff edge toward longer wavelengths at higher temperature. Laboratory tests demonstrate that when the BaF_2_ filter is heated to a temperature that exceeds 70 °C, the transmission at wavelengths that are shorter than 136 nm will be less than 1%.

The third is a low-pass detector for further suppressing the responses at wavelengths that exceed 180 nm. In the FUV range, the microchannel plate (MCP) photon-counting imaging detector is typically adopted. When the MCP is coated with cesium iodide (CsI) cathode on its light-sensitive surface, the quantum efficiency of the detector at wavelengths that exceed 180 nm will be substantially reduced. The detector body consists of an MCP stack, a preamplifier and a coding readout anode with wedge, stripe and zigzag styles. A flat MCP stack is adopted to match the flat focal plane of the optical system. The incoming photons strike the CsI photon cathode that has been deposited on the surface of the MCP, where they may eject photon-electrons, which are multiplied under high-voltage and deposited on the coding readout anode. The charge of the photon-electrons is proportionately divided among the wedge, stripe and zigzag electrodes. Then, the pulse signals are transferred to the electronic signals. Each of the three signals is amplified and converted to digital form prior to reaching the WAI position electronics. Each channel consists of a charge-sensitive amplifier, a pulse shaper, a peak holding unit and an A/D convert circuit and the three channels share a common pulse pile-up rejection unit.

In contrast to a charge-coupled device (CCD) detector, which has fixed physical pixels, the MCP detector has only equivalent pixels or virtual pixels. The location of a photon on the detector is calculated from the signals that are detected by the wedge, stripe and zigzag electrodes (see Supplementary Information for details). The virtual pixel size of the detector can be arbitrarily subdivided or binned by changing the scale factors in the calculations to obtain images at the designed resolution, which depends on the resolution of the optical system. For WAI, the pixel size has been set as 38.35 μm. By imaging the resolution chart directly without any optical system, the spatial resolution of the detector is measured to be ~77 μm, which corresponds to an angular resolution of ~0.22° since the focal length is 18.5 mm. Therefore, the angular resolution of WAI depends on the optical system.

### Sensitivity

The design of the WAI system sensitivity requires comprehensive consideration of the following factors: (1) the intensity of the dayglow and auroral emissions, (2) the transmission of the whole system (including mirrors, filters, and detectors), (3) the photon count rate of the detector, and (4) the data transmission rate of the satellite.

According to the observations from DMSP/SSUSI and TIMED/GUVI, the intensity of LBH auroral emissions varies from several hundred Rayleigh (1 Rayleigh = 10^6^/4*π* photon cm^−2^ s^−1^ sr^−1^) in nightside conditions to tens of thousands of Rayleigh in polar dayglow conditions. The efficiency of WAI should cover this intensity range. For a 100-Rayleigh emission, the number of counts of a pixel should be at least two during each accumulation period of 2.04 s to satisfy the Poisson statistics if we assume that the dark counts of the detector are negligible. The minimum sensitivity should be 0.01 counts s^−1^ Rayleigh^−1^ pixel^−1^ in 140–180 nm. For strong LBH emissions, e.g., 20,000 Rayleigh, a detector pixel can record at least 200 counts in 1 s. During severe storms, all the pixels of a camera might be filled with strong intensity; the maximum count rate for the detector should exceed 221,000 counts/s.

As discussed in the previous section, the pixel size is 0.8° × 0.8°, which corresponds to a solid angle of *ω* = 1.95 × 10^−4^ sr. Supposed the input aperture is *A* = 1 cm^2^ and the total response of the WAI system is *η* = 1. The equivalent sensitivity is *S*_*e*_ = *Aωη* × 10^6^/4*π* = 15.5 photons s^−1^ Rayleigh^−1^ for each square centimeter of the input aperture. The total transmission *η* is always less than one and is wavelength (*λ*)-dependent. For the WAI system, *η*(*λ*) depends on the reflectivity of the mirrors (*ρ*), the transmission of the filter (*τ*), and the quantum efficiency of the detector (*ε*), and *η*(*λ*) = *ρ*(*λ*) *τ*(*λ*) *ε*(*λ*). Therefore, the effective aperture, which equals the integral of *Aη*(*λ*) in the range from 140 to 180 nm, should be at least 0.00065 cm^2^.

The spectral response functions, namely, *ρ*(*λ*), *τ*(*λ*) and *ε*(*λ*), are measured using the high-precision far-ultraviolet spectral calibration system that was developed at CIOMP. This system is mainly composed of an EUV-FUV monochromator (Model XCT from McPherson, Inc.), a large vacuum chamber, a five-dimensional sample stage, a high-sensitivity photon-counting detector, and a high-stability vacuum FUV deuterium lamp. The spectral range of this system is from 50 to 300 nm with a spectral resolution of 0.4 nm and an accuracy of 1.5%. We measure the spectrum of the input beam (input signals) and, subsequently, measure the radiation that is reflected or transmitted by the element under test (the response signals). Finally, the functions *ρ*(*λ*), *τ*(*λ*), and *ε*(*λ*) can be obtained by dividing the response signals by the input signals.

**Fig. 5:**
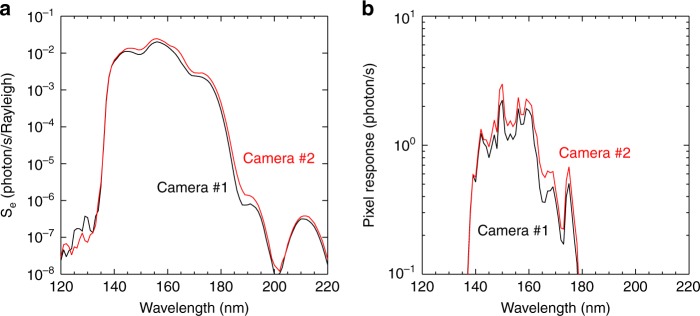
Spectral responses of WAI. **a** The sensitivities of the two cameras. **b** The pixel responses of the two cameras

Using the measured reflectivities of the four mirrors, the transmission of the filter (at 70 °C) and the quantum efficiency of the detector and setting *A* = 0.22 cm^2^ according to the optical design, we obtain the spectral sensitivity of the two cameras, as shown in Fig. 5a. Then, the sensitivities in the working band from 140 to 180 nm can be obtained by integrating their spectral sensitivities along the band, which are approximately 0.0145 counts s^−1^ Rayleigh^−1^ pixel^−1^ for camera #1 and 0.0160 counts s^−1^ Rayleigh^−1^ pixel^−1^ for camera #2. The uncertainties in the sensitivities are mainly due to the uncertainties in the calibration of the spectral responsivity of the optical elements. It is estimated that the uncertainty of the sensitivity is approximately 7.5%. Moreover, if we multiply the final sensitivity curve by the emission spectrum in Fig. 4, we obtain the pixel responses of the cameras to the auroral spectra in Fig. 5b. The out-of-band responses contribute less than 5% of the responses in 140–180 nm.

The spectral response is sensitive to the operating temperatures of WAI in orbit, such as the temperatures of the optical system and the filters. The designed operation temperature range is 15–45 °C in orbit and this is realized by the active thermal control system of WAI. A closed-loop control strategy is applied to the thermal controls of both the optical system and the filters with an accuracy of better than 2.5 °C^21^. Taken the spectral response at an ambient temperature of 25 °C and an operating temperature of 70 °C for the filters as a baseline, the change in the instrumental response as a function of the ambient temperatures is measured under laboratory calibrations (see Table S2 in Supplementary Information).

### WAI spatial resolution

**Fig. 6:**
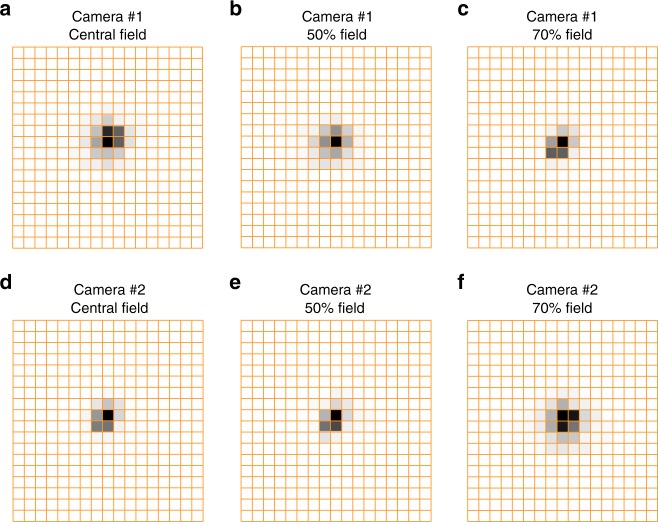
Spatial resolution test results. **a** Pinhole images of camera #1 at the central field. **b** Pinhole images of camera #1 at the 50% field. **c** Pinhole images of camera #1 at the 70% field. **d** Pinhole images of camera #2 at the central field. **e** Pinhole images of camera #2 at the 50% field. **f** Pinhole images of camera #2 at the 70% field. The orange grids represent the pixels of the detectors. Only part of the detector is displayed

The focal length of the optical system of WAI is 18.5 mm; testing its resolution by using a collimator with a resolution chart is not suitable. Instead, we have evaluated the spatial resolution of WAI by using a collimator with a pinhole. Meanwhile, because the depth of focus of the optical system exceeds 2 mm, we have also evaluated the spatial resolution by imaging a four-pinhole target at a distance of 120 times the focal length. At that distance, the imaging defocus was less than 0.15 mm; hence, the spot that was caused by defocus was much smaller than the pixel size and the test results could not be affected. The pinhole images of the two cameras are shown in Fig. 6. The field width at half maximum of the pinhole images is approximately 0.34°, which is better than 0.8°.

### FOV tests

The first step is to determine the FOV of a single camera. The camera was fixed on a two-dimensional gimbal and captured images of a pinhole, which was located at a distance of approximately 120 times the focal length from WAI. First, the pinhole was imaged at one edge of the 68° direction. Then, the pinhole was imaged at the other edge by rotating the gimbal. The rotation angle of the gimbal was read and recorded and the exact FOV in the 68° direction was obtained. The FOV in the 10° direction was obtained via the same procedure.

Then, the overlap angle between camera #1 and camera #2 was measured as 5.87°. Based on the above data, the combined FOV of WAI was computed, which was 134.43°. Finally, the scanning range of WAI along the track was measured, which was approximately 123.03°. Therefore, the actual total FOV of WAI is 134.43° × 133.24°.

### Geometrical calibration

WAI uses photon-counting detectors, which exhibit obvious imaging nonlinearity on its edge. The optical systems also have large geometric distortion due to their large FOV. These two factors cause high nonlinearity in WAI’s image plane; therefore, it is necessary to correct the nonlinearities in the detectors and the optical systems. The first step is to correct the detector distortion and the second step is to correct the camera distortion that is produced by the optical systems.

The detector’s imaging nonlinearity was sampled by imaging an equally spaced pinhole array. Due to the detector’s imaging nonlinearity, the actual positions of the pinholes in the images deviated from their ideal positions. The ideal positions of the pinholes in the images were already known based on the structure of the pinhole array. By subtracting the ideal positions from the deviated positions of the pinholes, a look-up table was established. By querying the look-up table, the imaging distortion of the detector can be corrected.

**Fig. 7:**
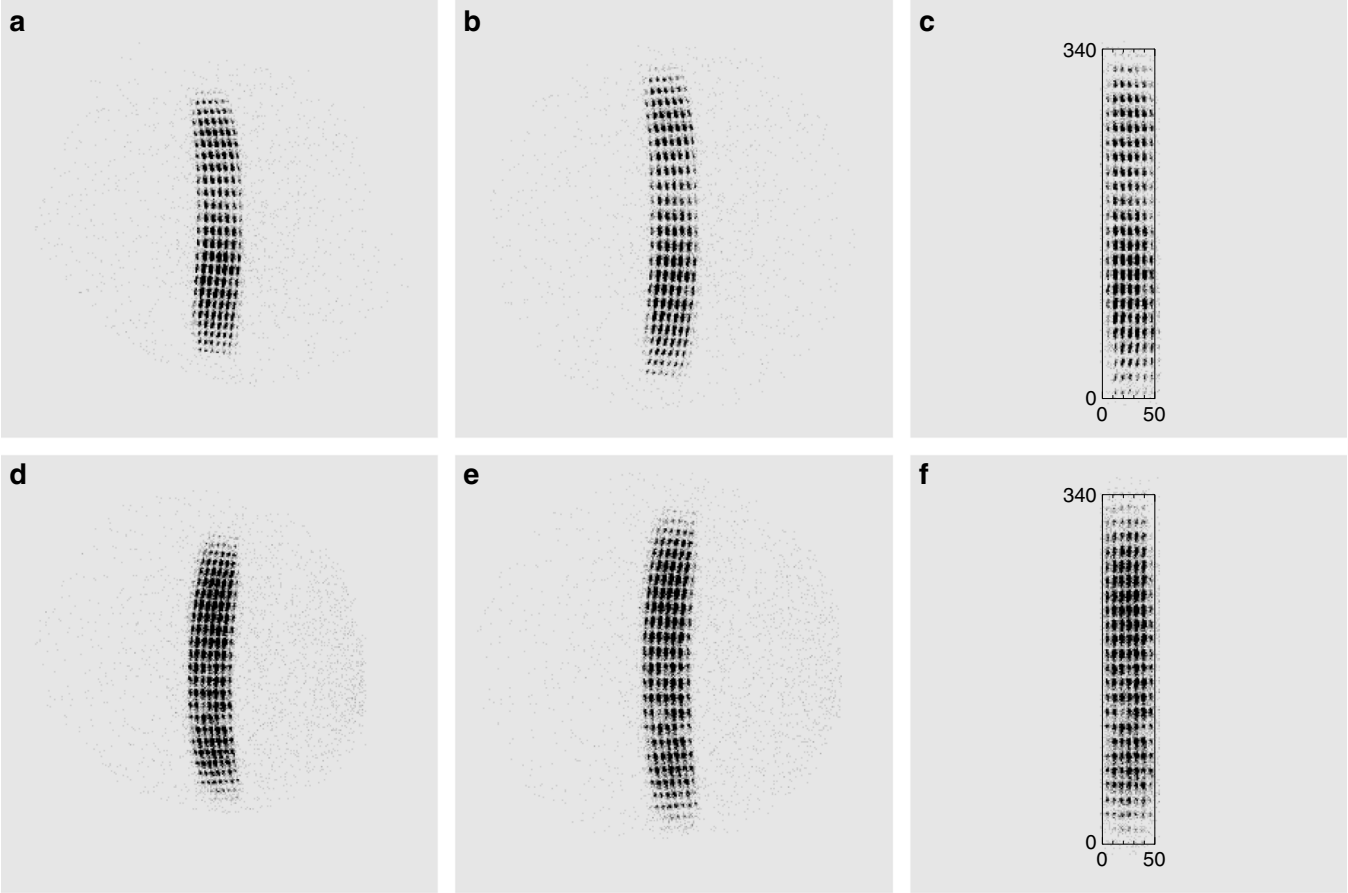
Corrections of the geometrical nonlinearities for the two cameras. **a** An original image of the equally spaced pinhole array of camera #1. **b** The image of camera #1 with the detector nonlinearity corrected. **c** The final image of camera #1 with all geometrical nonlinearities corrected. **d** An original image of the equally spaced pinhole array of camera #2. **e** The image of camera #2 with the detector nonlinearities corrected. **f** The final image of camera #2 with all geometrical nonlinearities corrected. The coordinates in (**c**) and (**f**) correspond to the FOVs of the cameras. The equivalent number of pixels are 340 and 50 for the FOVs of 68° and 10°, respectively

After system integration was completed, WAI imaged the pinhole array to sample the residual imaging nonlinearity of the cameras that was produced by the optical system. The distorted images were compared with the ideal images of the pinhole array to obtain a new look-up table, which was used to correct the residual distortion. The calibrated images are shown in Fig. 7. Figure 7a, d shows the original images, which have nonlinearities that are produced by both the detector and the optical system. Figure 7b, e shows the images with the detector nonlinearities corrected. Figure 7c, f shows the final images with all the geometrical nonlinearities corrected. After detector-level and system-level corrections, the geometrical nonlinearity of WAI is less than 2%.

### Flat field

**Fig. 8:**
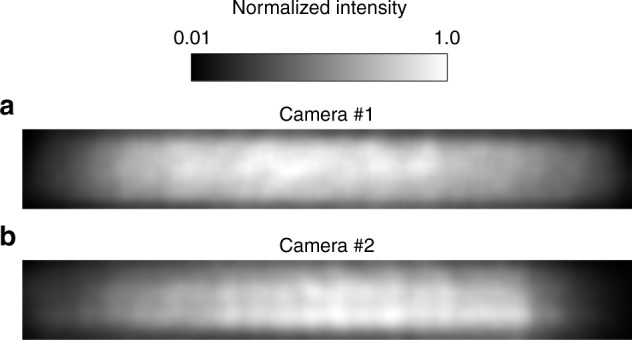
Flat-field images. **a** A normalized flat-field image for camera #1. **b** A normalized flat-field image for camera #2

The purpose of the flat field is to calibrate the nonuniformity in the intensity response among various positions of the image plane. This nonuniformity could be caused by the vignetting of the optical system and the nonuniform reflectivity of the mirrors, the transmission of the filters and the quantum efficiency of the detectors. Flat-field calibration is typically implemented by imaging a uniform target, which must cover the full aperture and the full FOV of the instrument. WAI operates in the FUV band and there is no Lambertian extended uniform source in this wavelength that is sufficiently large to cover WAI’s full FOV. We constructed a target by using an FUV deuterium lamp to illuminate an MgF_2_ diffuser. This target can be viewed as a Lambertian uniform target in approximately 20° FOV. Only the uniform region of the target was used during the calibration process. Both the cameras and the diffuser were placed in a vacuum chamber. The cameras were rotated and imaged the uniform portion of the diffuser target at various parts of their FOVs. The cameras acquired images with the same exposure time. Then, all the images were combined to form a full-FOV flat-field image. The results are shown in Fig. 8. The calibration error was mainly caused by the instability of the light source, the uniformity of the illumination of the MgF_2_ diffuser, and the Lambertian error. The light source instability was better than 1%. The uniformity of the 20° portion of the diffuser was better than 5% and its Lambertian error was less than 6%. The total error in the flat-field calibration was approximately 7.9%.

### Data processing algorithm

The data processing algorithm for WAI consists of the following steps: geometric correction, photometric correction, LOS projection and disk image generation. These steps will be briefly introduced in this paper and the algorithm will be described in detail in the Supplementary Information.

The first step is to calculate the location of an incident photon on the detector. Then, the geometric distortions that are caused by the detector and the optical system are corrected. The photometric correction of the images involves three substeps: The first substep is to correct the nonlinearity of the response of the detector, the second substep is to correct the differences in the response of the detector at various temperatures, and the final substep is flat-field correction. Then, each photon that is recorded in each pixel must be traced back to its source in the auroral oval, namely, the pixels must be mapped onto a reference sphere (e.g., 110 km). Finally, the images from the two cameras are merged to generate a complete disk image on the reference sphere.

## Supplementary information

Supplementary Information-WAI Data Processing Algorithm

## References

[1] Meier RR (1991). Ultraviolet spectroscopy and remote sensing of the upper atmosphere. Space Sci. Rev..

[2] Dashkevich ZV, Sergienko TI, Ivanov VE (1993). The Lyman–Birge–Hopfield bands in aurora. Planet. Space Sci..

[3] Germany GA (1994). Use of FUV auroral emissions as diagnostic indicators. J. Geophys. Res. Space Phys..

[4] Germany GA (1994). Determination of ionospheric conductivities from FUV auroral emissions. J. Geophys. Res. Space Phys..

[5] Frank LA (1981). Global auroral imaging instrumentation for the Dynamics Explorer mission. Space Sci. Instrum..

[6] Mende SB (2000). Far ultraviolet imaging from the IMAGE spacecraft. 1. System design. Space Sci. Rev..

[7] Mende SB (2000). Far ultraviolet imaging from the IMAGE spacecraft. 2. Wideband FUV imaging. Space Sci. Rev..

[8] Mende SB (2000). Far ultraviolet imaging from the IMAGE spacecraft. 3. Spectral imaging of Lyman-α and OI 135.6 nm. Space Sci. Rev..

[9] Torr MR (1995). A far ultraviolet imager for the international solar-terrestrial physics mission. Space Sci. Rev..

[10] Christensen, A. B. et al. Global ultraviolet imager (GUVI) for the NASA thermosphere–ionosphere–mesosphere energetics and dynamics (TIMED) mission. *Proceedings of SPIE 2266, Optical Spectroscopic Techniques and Instrumentation for Atmospheric and Space Research* (SPIE, San Diego, CA, United States, 1994).

[11] Paxton, L. J. et al. Special sensor ultraviolet spectrographic imager: an instrument description. *Proceedings of the Instrumentation for Planetary and Terrestrial Atmospheric Remote Sensing* (SPIE, San Diego, CA, United States, 1992).

[12] Strickland DJ, Evans JS, Paxton LJ (1995). Satellite remote sensing of thermospheric O/N_2_ and solar EUV: 1. Theory. J. Geophys. Res. Space Phys..

[13] Johnstone AD (1978). Pulsating aurora. Nature.

[14] Frank LA (1986). The theta aurora. J. Geophys. Res. Space Phys..

[15] Farrugia CJ, Sandholt PE, Burlaga LF (1994). Auroral activity associated with Kelvin–Helmholtz instability at the inner edge of the low-latitude boundary layer. J. Geophys. Res. Space Phys..

[16] Laundal KM, Østgaard N (2009). Asymmetric auroral intensities in the Earth’s northern and southern hemispheres. Nature.

[17] Han DS (2016). Throat aurora: the ionospheric signature of magnetosheath particles penetrating into the magnetosphere. Geophys. Res. Lett..

[18] Tu CY (2008). Space weather explorer—the KuaFu mission. Adv. Space Res..

[19] Laikin, M. *Lens Design* 4th edn (CRC Press, Boca Raton, FL, 2007).

[20] Christensen AB (2003). Initial observations with the Global Ultraviolet Imager (GUVI) in the NASA TIMED satellite mission. J. Geophys. Res. Space Phys..

[21] Yang HB (2014). Thermal design and verification of transmission filter for wide angle aurora imager. Opt. Precis. Eng..

