# Supplementary material for: Wide-field auroral imager onboard the Fengyun satellite
Source: Light Sci Appl. 2019 May 22;8:47. doi: 10.1038/s41377-019-0157-7 (PMC6529440; doi:10.1038/s41377-019-0157-7)
Supplement: Supplementary file 1 — Supplementary Information-WAI Data Processing Algorithm [file 41377_2019_157_MOESM1_ESM.docx]

Supplementary Information

**Wide-field Auroral Imager Onboard the Fengyun Satellite**

^*1,2^Xiao-Xin Zhang, ^*3,4^Bo Chen, ^*5,6^Fei He, ^3,4^Ke-Fei Song, ^3,4^Ling-Ping He, ^3,4^Shi-Jie Liu, ^3^Quan-Feng Guo, ^1,2^Jia-Wei Li, ^3^Xiao-Dong Wang, ^3^Hong-Ji Zhang, ^3^Hai-Feng Wang, ^3^Zhen-Wei Han, ^3^Liang Sun, ^3^Pei-Jie Zhang, ^3^Shuang Dai, ^3^Guang-Xing Ding, ^3^Li-Heng Chen, ^3^Zhong-Su Wang, ^3^Guang-Wei Shi, ^3^Xin Zhang, ^1,2^Chao Yu, ^1^Zhong-Dong Yang, ^1^Peng Zhang, ^1^Jin-Song Wang

^1^National Satellite Meteorological Center, China Meteorological Administration, Beijing, China.

^2^KeyLaboratory of Space Weather, National Center for Space Weather, China Meteorological Administration, Beijing, China

^3^Changchun Institute of Optics, Fine Mechanics and Physics, Chinese Academy of Sciences, Changchun, China.

^4^State Key Laboratory of Applied Optics, Chinese Academy of Sciences, Changchun, China.

^5^Key Laboratory of Earth and Planetary Physics, Institute of Geology and Geophysics, Chinese Academy of Sciences, Beijing, China.

^6^Institutions of Earth Science, Chinese Academy of Sciences, Beijing, China.

*These authors contributed equally to this work.

Corresponding authors: Xiao-Xin Zhang (xxzhang@cma.gov.cn), Bo Chen (chenb@ciomp.ac.cn).

**I. Overview of the data processing algorithm**

The information flow chart of the data processing algorithm (DPA) is shown in Fig. S1. The original data packets (Level-0) are first loaded from the database into the DPA. The packets are divided into single frames according to the beginning identifier and ending identifier prior to the following steps. Then the physical parameters are decoded according to the data format pre-defined. Detailed descriptions of the steps 3-7 are as follows.

**
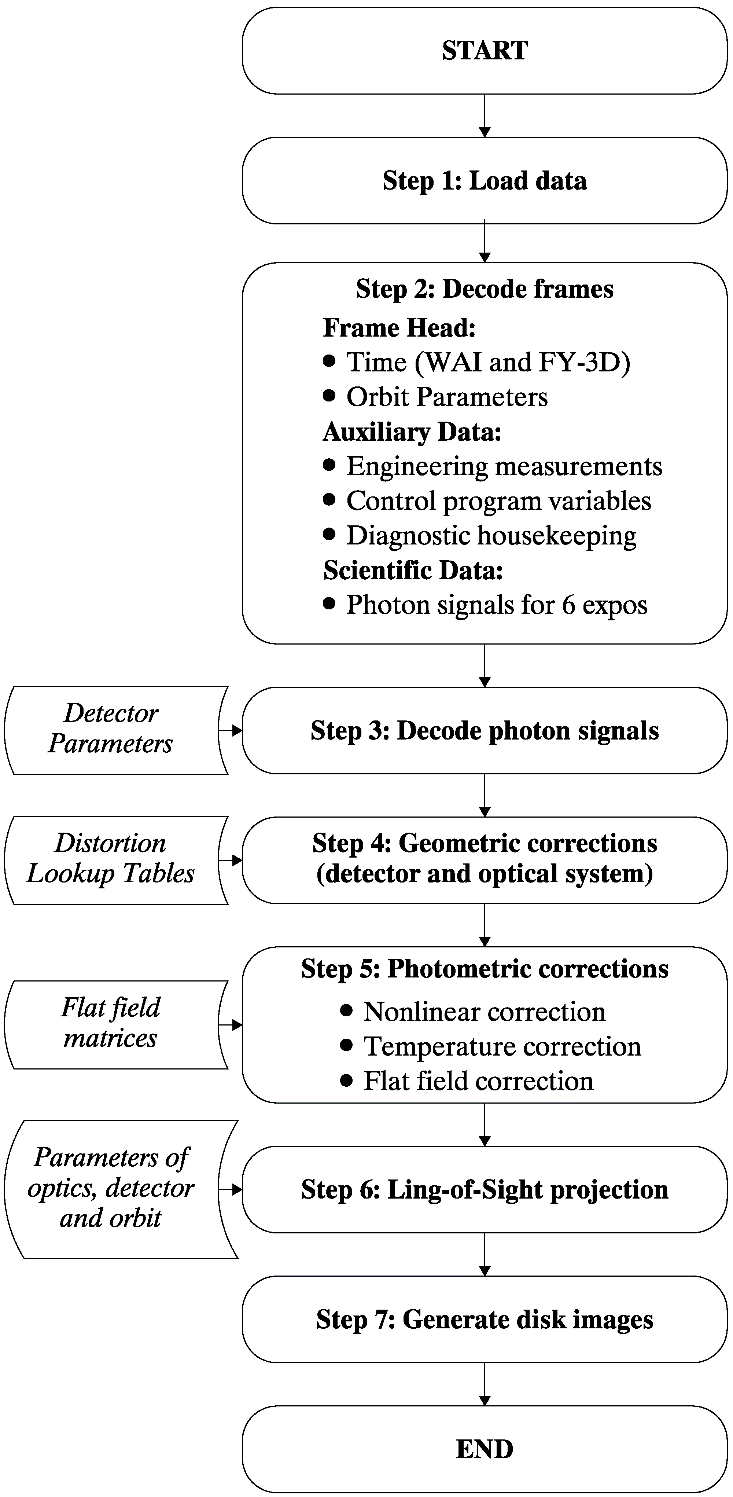
**

**Fig.S1 Flowchart of the WAI DPA.**

**II. Geometric correction**

The photons arrive the detector are transformed into photoelectric signals by the MCP stack and then readout by the wedge and strip anode. The charge is proportionately divided among the wedge, strip and zigzag electrodes, and then the signals (W, S, and Z) are transferred to the detector electronics. Each of the three signals is amplified and converted to digital form and saved to the data packets. The X, Y photon positions of a photon in the plane of the sensor are calculated with the following formula:

$\begin{matrix} X=K\times a_{1}\frac{Q_{S}}{Q_{S}+Q_{W}+Q_{Z}}-x_{0} \\ Y=K\times a_{2}\frac{Q_{W}}{Q_{S}+Q_{W}+Q_{Z}}-y_{0} \end{matrix}$ (1)

where *Q*_S_, *Q*_W_ and *Q*_Z_ are charge digitized on the wedge, strip, and zigzag electrodes respectively, the parameters *K*, *a*_1_ and *a*_2_ are scale factors, and *x*_0_ and *y*_0_ are shift factors that transform the normalized charge values to a cartesian detector coordinate system. Then the geometric distortion generated by the detector and the optical system are corrected using table lookup. For each exposure, we can get a two-dimensional matrix of image (**I**_0_, **I**_00_ for detector #1 and **I**_01_ for detector #2) in the plane of the sensor. Each element of the matrix represents a ‘pixel’ in the focal plane without geometric distortion. The value of each pixel represents the photons recorded by the detector in the cone angle defined by the detector pixel and the optical system. According to the FOV, focal length and angular resolution of WAI, the scale factor *K* has been chosen as 3000 to make the pixel size to be 38.35 μm, and the size of the image matrix is 667×87 for the FOV of 68°×10°. The optical axis is located at the center of the matrix. Then the image **I**_0_ is transferred to the next step for photometric correction.

**III. Photometric correction**

The photometric correction of the images includes three sub-steps. The first step is to correct the nonlinearity of the response of the detector. The incident photons cannot be wholly recorded by the electronics especially when the count rate is large. It is assumed that the missing counting is uniform in the detector plane since all the photons are recorded though the WSZ electrodes. The correction coefficients for different numbers of recorded photons have been determined in laboratory calibration and listed in Table S1. Since the electronics of the two detectors are identical, their correction coefficients are the same.

**Table S1.** Correction coefficients for detector nonlinearity.

| **Recorded Photon Numbers** | **Correction Coefficient** |
| --- | --- |
| 0 | 1.0000 |
| 24052 | 1.0465 |
| 53285 | 1.0633 |
| 90491 | 1.1095 |
| 185001 | 1.1700 |
| 235846 | 1.2492 |
| 287918 | 1.3365 |

To correct this nonlinearity, the recorded photon numbers in the auxiliary data of the frame are loaded, then linear interpolations are done to get the coefficients for the two detectors (*c*_n0_ for detector #1 and *c*_n1_ for detector #2). Then the image **I**_0_ is corrected by the coefficient to get a new image **I**_1_, i.e., **I**_10_=*c*_n0_**I**_00_ and **I**_11_=*c*_n1_**I**_01_.

The second step is to correct the differences of the responses of the detectors at different temperature. Take the responses of the detectors at 25 ℃ as a reference, the responses at other temperatures ranging from 15 ℃ to 45 ℃ are measured in laboratory calibrations and the correction coefficients for the two detectors are listed in Table S2.

**Table S2.** Correction coefficients for temperature

| **Temperature (℃)** | **Detector #1** | **Detector #2** |
| --- | --- | --- |
| 15.0 | 0.8363 | 0.9888 |
| 17.5 | 0.8772 | 0.9916 |
| 20.0 | 0.9181 | 0.9944 |
| 22.5 | 0.9591 | 0.9972 |
| 25.0 | 1.0000 | 1.0000 |
| 27.5 | 1.0491 | 1.0060 |
| 30.0 | 1.0983 | 1.0120 |
| 32.5 | 1.1474 | 1.0179 |
| 35.0 | 1.1965 | 1.0239 |
| 37.5 | 1.1927 | 1.0141 |
| 40.0 | 1.1889 | 1.0042 |
| 42.5 | 1.1851 | 0.9944 |
| 45.0 | 1.1814 | 0.9845 |

To correct the response difference at different temperature, the detector temperatures in the auxiliary data of the frame are loaded, then linear interpolations are done to get the coefficients for the two detectors (*c*_t0_ for detector #1 and *c*_t1_ for detector #2). Then the image **I**_1_ is corrected by the coefficient to get a new image **I**_2_, i.e., **I**_20_=*c*_t0_**I**_10_ and **I**_21_=*c*_t1_**I**_11_.

The final step is flat field correction. Responses of the optical system at different angle are different due to the vignetting of the optics and the small changes of the reflectivity of the mirrors at different incident angles. The nonuniformity in the focal plane can be measured using uniform beam as demonstrated in the paper. Take the center of the FOV as a reference, a normalized matrix can be generated for the two detectors (**M**_0_ and **M**_1_). The format of the two matrices are the same the scientific images. The final scientific images can be obtained as **I**_30_=**M**_0_**I**_20_/(*S*_0_×Δ*t*) and **I**_31_=**M**_1_**I**_21_/(*S*_0_×Δ*t*) in unit of Rayleigh (1 Rayleigh=10^6^/4π photon cm^-2^ s^-1^ sr^-1^, 1 kR=1000 Rayleigh), where *S*_0_ is the sensitivity and Δ*t* is the accumulation time for each exposure. These images are now ready for line-of-sight (LOS) projections and generation of disk images.

**IV. LOS projection**

The photons recorded in each pixel must be traced back to its source in the auroral oval, that is the pixels must be mapped onto a reference sphere (i.e., 110 km). The first step to map a pixel in the detector plane to space is to establish a series of transformation matrices from the detector to space. For WAI, the coordinate systems used are defined in Table S3 and Fig. S2. The flowchart of the coordinate transformation is shown in Fig.S3. Some of the transformation matrices are determined during laboratory tests and calibrations, i.e., **P**_1_ and **P**_3_, others should be calculated based on time and location of imaging. **P**_2_ has two forms. The first form is for nadir mode and is calibrated in laboratory. The second form is for scanning mode and should be updated for each exposure based on the first form according to the position of the rotator. **P**_4_-**P**_8_ are calculated according to the position, velocity, and posture of the satellite and should also be updated for each exposure.

**Table S3.** Definition of coordinate systems for WAI

| **System** | **Definition of axes** |
| --- | --- |
| Image Coordinate System, ICS ^a^ | Z = Optical axis  Y = Along the 68° FOV |
| Head Coordinate System, HCS | Z = Normal of the prism at the camera that along the optical axis  Y = Normal of the prism at the camera that along the 68° FOV |
| Camera Coordinate System, CCS | Z = Normal of the prism at the left arm that along the optical axis  Y = Normal of the prism at the left arm that along the 68° FOV |
| Satellite Coordinate System, SCS | X = Along the direction of launch  Z = Perpendicular to the surface facing the Earth in orbit |
| Orbit Coordinate System, OCS | X = Velocity of the satellite  Z = From the satellite to the Earth’s center |
| Orbit Primary System, OPS ^b^ | X = From Earth’s center to the first location of the satellite **V**_1_  Z = The normal of the great circle defined by the first location and the last location of the satellite **V**_2_, and **Z**=**V**_1_×**V**_2_ |
| Geographic, GEO | *X* = Intersection of Greenwich meridian and geographic equator  *Z* = Geographic North Pole |
| Geomagnetic, MAG | *Y* = Intersection between geographic equator and the geographic meridian 90° East of the meridian containing the dipole axis.  Z = Dipole axis |
| Note: Only two axes have to be defined; the third axis completes a right-handed Cartesian triad. Definitions of ICS, HCS, CCS are shown in Figure 3.  ^a^ Each camera has its own ICS.  ^b^ OPS is just used to generate disk images. The satellite positions of the frames used to generate the disk image are used to define the OPS. | |

**
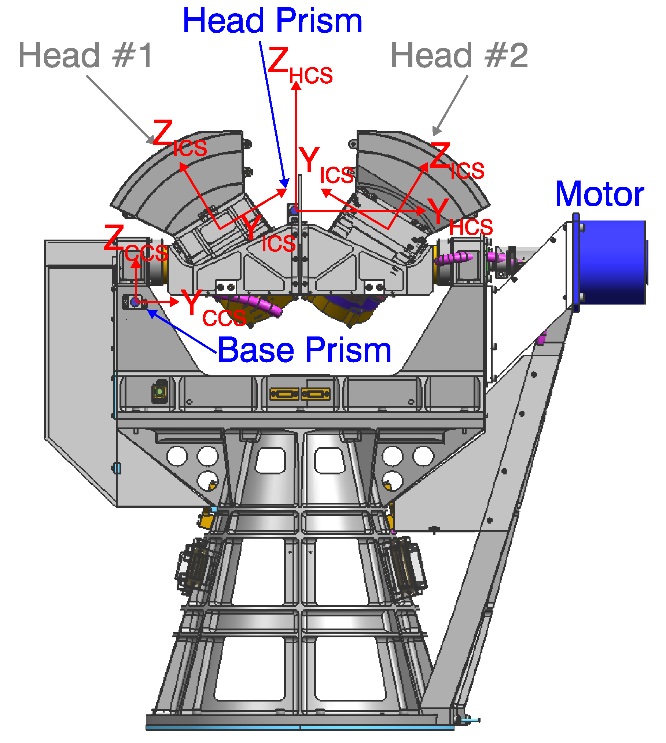
**

**Fig.S2 Definition of the coordinate systems for WAI.**


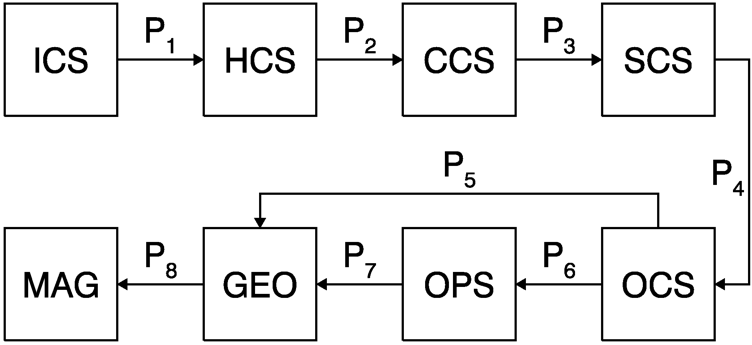


**Fig.S3 Flowchart of the coordinate transformation for the WAI DPA.**

The LOS projection begins with calculation of the LOS vector in ICS (**LOS**_ICS_ = [*v*_icsx_, *v*_icsy_, *v*_icsz_]). In each exposure image, the detector pixel size is *δ* on the focal plane with a focal length of *f*, and then the pitch (*ρ*) and azimuthal (*ϕ*) angle for the **LOS**_ICS_ can be calculated by

 (2)

Then, we can get

 (3)

Finally, the LOS vector in GEO (**LOS**_GEO_ = [*v*_smx_, *v*_smy_, *v*_smz_]) can be calculated through

$\mathbf{LOS}_{\text{GEO}}=\mathbf{T}_{\text{ICSGEO}}\mathbf{LOS}_{\text{ICS}}$ (4)

where the transformation matrix **T**_ICSGEO_ = **P**_5_**P**_4_**P**_3_**P**_2_**P**_1_. After laboratory calibration, the matrices **P**_1_ are

$\mathbf{P}_{1}=\left[\begin{matrix} 0.99906055 & 0.00000000 & \text{-0.04333603} \\ \text{-0.02298335} & 0.84777756 & \text{-0.52985373} \\ 0.03673931 & 0.53035197 & 0.84698112 \end{matrix}\right]$ (5)

for detector #1, and

$\mathbf{P}_{1}=\left[\begin{matrix} 0.99934733 & 0.00000000 & 0.03612372 \\ \text{-0.01966201} & 0.83889307 & 0.54394101 \\ \text{-0.03030394} & \text{-0.54429626} & 0.83834555 \end{matrix}\right]$(6)

for detector #2, respectively.

When the cameras are absolutely pointed to nadir (+Z axis of CCS), the matrix **P**_2_ is a unit matrix. For nadir mode, however, there is a pointing error of *δ*=0.0494° for ascending orbits and *δ*=0.0837° for descending orbits due to the different control strategies of the motor. Therefore, **P**_2_ is

$\mathbf{P}_{2}=\left[\begin{matrix} \cos\delta& 0 & \sin\delta\\ 0 & 1 & 0 \\ -\sin\delta& 0 & \cos\delta\end{matrix}\right]$ (7)

for nadir mode. For scanning mode, the angular position of the camera prism is

$\theta=60^{\circ}-\left[(j-1)\times(N\times\Delta t+dt)+(i-0.5)\times\Delta t\right]\times v_{\mathrm{scan}}$ (8)

for backward sweeping and

$\theta=-60^{\circ}+\left[(j-1)\times(N\times\Delta t+dt)+(i-0.5)\times\Delta t\right]\times v_{\mathrm{scan}}$ (9)

for forward sweeping, where *N* is number of exposures for a frame (*N*=2, 4, 6, 8, or 10), Δ*t*=0.34 s is the accumulation time for each exposure, *dt* is the waiting time during adjacent frames (*dt* =7.46 s, 7.40 s, 7.46 s, 7.66 s, or 6.91 s, determined by *N*), *v*_scan_=1.125°/s is the rotating speed of the camera, and *i* and *j* are indices of the exposure and frame during each sweeping cycle, respectively. Then **P**_2_ is determined by

$\mathbf{P}_{2}=\left[\begin{matrix} \cos\theta& 0 & \sin\theta\\ 0 & 1 & 0 \\ -\sin\theta& 0 & \cos\theta\end{matrix}\right]$. (10)

The matrix **P**_3_ for the transformation from CCS to SCS is

$\mathbf{P}_{3}\text{=}\left[\begin{matrix} 0.99999965 & \text{-0.00071752} & 0.00042178 \\ 0.00071752 & 0.99999974 & \text{-0.00003878} \\ \text{-0.00042178} & 0.00003393 & 0.99999991 \end{matrix}\right]$ (11)

Until now, we have established the coordinate transformation chain from a detector pixel to the satellite. Once we have known the position (in GEO), velocity (in GEO), and posture (in OCS) information of the satellite, the transformation matrices **P**_4_, **P**_5_, and **P**_8_ can be determined and the LOS vector can be transformed to GEO. Then the latitude/longitude (both GEO and MAG) of the intersection of the LOS to the reference sphere at 110 km height can be calculated for each pixel in addition to the limb pixels. Now, the LOS projection procedure is finished for the image during each exposure. In the following step, images from multiple frames will be used to generate disk images of the aurora or ionosphere.

**V. Disk image**

To merge the images from the two cameras to generate a complete disk image on the reference sphere, it is better to establish a coordinate system on this sphere. Since the shape and scope of the disk image is decided by how many frames used, the orbit information of these frames is used to define the OPS. Before generating the latitude/longitude grid for the disk image, the definition of the OPS and transformation matrix **P**_7_ should be calculated. The definition of OPS is shown in Fig. S4. The methods to establish OPS in GEO are the same for both scanning mode and nadir mode.


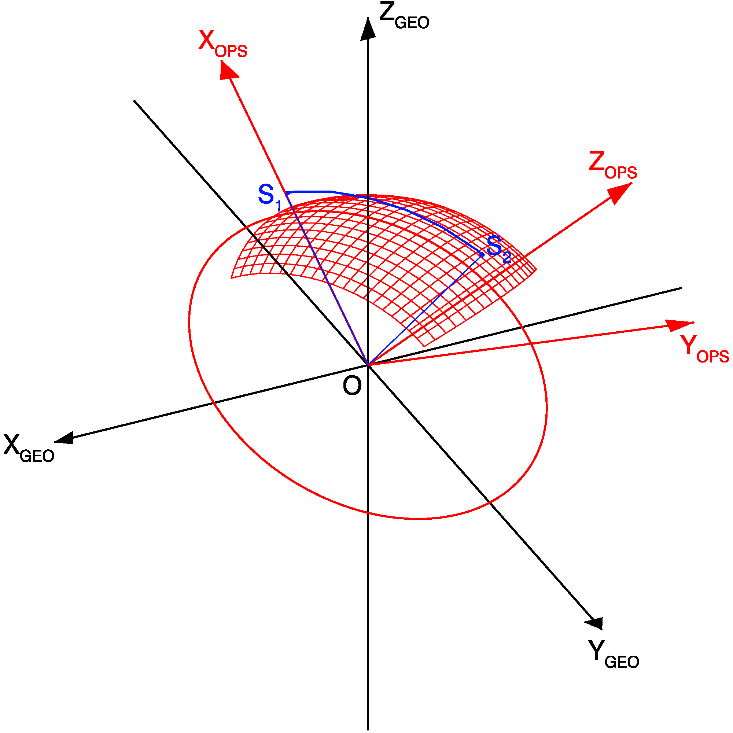


**Fig. S4 Definition of OPS and the grids for disk images.** Point O represents Earth’s center. The thick red circle represents the great circle in the xy-plane of OPS. The red grids represent the latitudinal/longitudinal grids in the reference sphere of 110 km height. The grids are symmetric to the meridian plane defined by the red circle.

Suppose that the observation data from satellite position S_1_ to S_2_ will be used to construct the disk image of the aurora. Let vectors **V**_1_=OS_1_ and **V**_2_=OS_2_. The x axis of OPS (**X**_OPS_=[*x*_xops_, *y*_xops_, *z*_xops_]) is **X**_OPS_=**V**_1_. The z axis of OPS (**Z**_OPS_=[*x*_zops_, *y*_zops_, *z*_zops_]) is perpendicular to the great circle defined by OS_1_ and OS_2_, and **Z**_OPS_ = **V**_1_ × **V**_2_. Then the y axis of OPS (**Y**_OPS_=[*x*_yops_, *y*_yops_, *z*_yops_]) is determined by the right-hand law. All the unit vectors of **X**_OPS_, **Y**_OPS_, and **Z**_OPS_ are defined in GEO. So, the elements of **P**_7_ can be determined by the direction cosine of the axes of OPS in GEO as

$\mathbf{P}_{7}=\left[\begin{matrix} x_{\text{xops}} & x_{\text{yops}} & x_{\text{zops}} \\ y_{\text{xops}} & y_{\text{yops}} & y_{\text{zops}} \\ z_{\text{xops}} & z_{\text{yops}} & z_{\text{zops}} \end{matrix}\right]$ (12)

Now, we can define the grids for the disk images in OPS and then transformed to GEO/MAG. For a 10 km by 10 km pixel in the reference sphere at 110 km height, the corresponding angle relative to the Earth’s center is 0.088°. So, the grid will be established with intervals of 0.088°. Construction of the grid for scanning mode and nadir mode is slightly different.

In the cross-track direction (Fig.S5a), the FOV is 130° as shown by the orange lines. Only the LOS that intersects with the reference sphere is considered in construction of the disk image as shown by the dashed orange lines in Fig. S5a. Suppose the altitude of FY-3D orbit is *H*, the radius of the reference sphere is *R*_110_, and the Earth radii is *R*_E_, the angle *θ*_ct_ can be calculated with

$\theta_{\text{ct}}=\text{acos}\left(\frac{R_{\text{r}}}{R_{\text{E}}\text{+}H}\right)$ (13)

Since the orbit plane does not strictly lie in the great circle of OPS, to ensure the completeness of the imaging data, the angle *θ*_ct_ should be slightly enlarged. The tilt angle of FY-3D is ~99°. For nadir mode in the low magnetic latitudes from -60° to 60°, the maximum angle between the orbit plane and the great circle is less than 3.0°. For scanning mode, this angle can be neglected, and we just need to add two grid points in the two sides. Therefore, the number of grid points cross-track are *N*_ct_ = [2(*θ*_ct_+3.0)/0.088]+1 for nadir mode and *N*_ct_ = [2*θ*_ct_/0.088]+5 for scanning mode and make sure the grid is symmetric to the xy plane of OPS ([] represents round-off). The equivalent latitude in OPS begins from –(*N*_ct_-1)×0.044 to (*N*_ct_-1)×0.044.

The grid dimension along-track depends on operation modes. For nadir mode (Fig. S5b), the grid dimension along-track is decided by the number of frames used, e.g., from Q_1_ to Q_2_ in Fig. S5b. The azimuthal angle in the xy plane of OPS includes two parts. The first part is the angle between OQ_1_ and OQ_2_, which can be calculated from the orbit parameters. The second part is the generated by the half FOV along-track, i.e. the angle between the blue and orange dashed lines which can be easily calculated as

$\theta_{\text{at}2}=\text{-asin}\left(\frac{\text{R}_{\text{E}}\text{+H}}{\text{R}_{\text{r}}}\sin5^{\circ}\right)\text{+}5^{\circ}$ (14)

and

$\theta_{\text{at}1}=\text{acos}\left(\frac{\vec{\mathbf{Q}}_{\mathbf{1}}\bullet\vec{\mathbf{Q}}_{\mathbf{2}}}{\left|\vec{\mathbf{Q}}_{\mathbf{1}}\right|\left|\vec{\mathbf{Q}}_{\mathbf{2}}\right|}\right)\text{+asin}\left(\frac{R_{\text{E}}\text{+}H}{R_{\text{r}}}\sin5^{\circ}\right)-5^{\circ}$. (15)

For scanning mode, the sweeping range is from -60° to 60°, as shown in Fig.S5c-S5d. In the case of forward scanning in Fig. S5c, it can be calculated as

$\theta_{\text{at}2}=\theta_{\text{ct}},\theta_{\text{at}1}=\text{acos}\left(\frac{\vec{\mathbf{Q}}_{\mathbf{1}}\bullet\vec{\mathbf{Q}}_{\mathbf{2}}}{\left|\vec{\mathbf{Q}}_{\mathbf{1}}\right|\left|\vec{\mathbf{Q}}_{\mathbf{2}}\right|}\right)\text{+}\theta_{\text{ct}}$. (16)

In the case of backward scanning in Fig.6d, we can get

$\theta_{\text{at}2}=\theta_{\text{ct}}\text{-acos}\left(\frac{\vec{\mathbf{Q}}_{\mathbf{1}}\bullet\vec{\mathbf{Q}}_{\mathbf{2}}}{\left|\vec{\mathbf{Q}}_{\mathbf{1}}\right|\left|\vec{\mathbf{Q}}_{\mathbf{2}}\right|}\right),\theta_{\text{at}1}=\text{+}\theta_{\text{ct}}$. (17)

Then, the number of grid points cross-track is *N*_al_ =[*θ*_at1_/0.088]+[*θ*_at2_/0.088]+1 and the grid begin at the azimuthal angle of –[*θ*_at2_/0.088]×0.088.


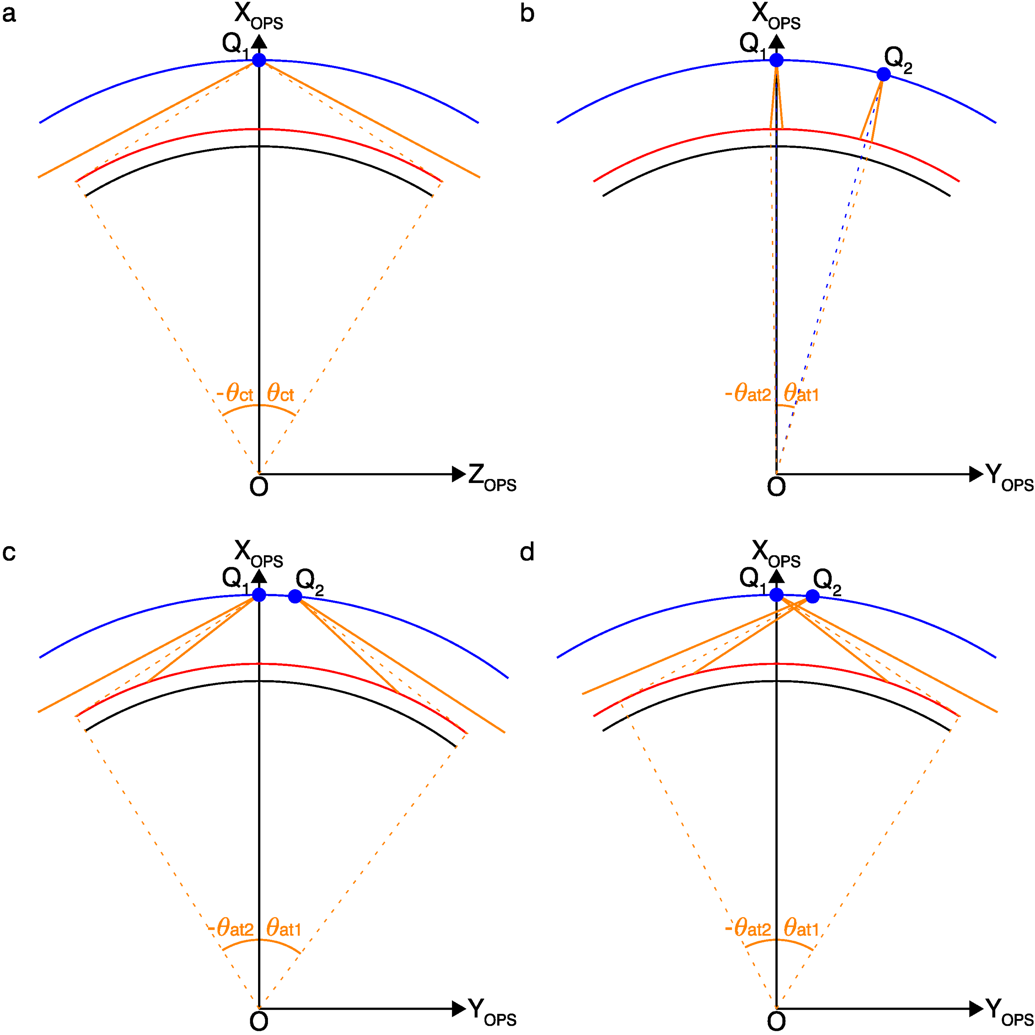


**Fig. S5 Determination of the boundary of grid for different modes. a** Cross-track view. **b** Along-track view for nadir mode. **c** Along-track view for forward scanning mode. **d** Along-track view for backward scanning mode. The blue, red and black arcs represent the satellite orbit, reference sphere, and the Earth surface, respectively. The orange thick solid lines show the FOV cross-track or along-track.

Finally, the coordinates of the grid points and the grid center can be calculated in OPS and transformed to GEO/MAG.

Each LOS from either detector is then mapped into the grid to generate a disk image. It is noted that, most of the pixels are not in a nadir observing perspective since WAI has a large FOV and large sweep range. A preliminary method is proposed to eliminate this effect. The modified Atmospheric Ultraviolet Radiance Integrated Code is used to calculate the correction coefficient. This method was also adopted in the process of the observation data of GUVI and SSUSI. The pixel value of the disk image is calculated by averaging all the detector pixels mapped into that grid. Then the pixel values of the overlap region of the two cameras are summed respectively to get normalization coefficients to normalize the intensity of the two cameras.
